# Risk factors of osteonecrosis in patients with systemic lupus erythematosus: a meta-analysis

**DOI:** 10.3389/fmed.2025.1694721

**Published:** 2025-10-17

**Authors:** Xiang Zhou, Yijie Chai

**Affiliations:** ^1^Department of Internal Medicine, Xianju Hospital of Traditional Chinese Medicine, Taizhou, Zhejiang, China; ^2^Emergency Department, Xianju Hospital of Traditional Chinese Medicine, Taizhou, Zhejiang, China

**Keywords:** systemic lupus erythematosus, osteonecrosis, risk factors, meta-analysis, systematic review

## Abstract

**Background:**

This study aimed to explore risk factors for osteonecrosis in patients with systemic lupus erythematosus by meta-analysis.

**Methods:**

PubMed, Embase, Web of Science, and Cochrane Library were searched for case–control/cohort studies on the occurrence of osteonecrosis in patients with systemic lupus erythematosus from the time the database was created until 1 December 2024. Data were analyzed using Stata 15.0, and the quality of the included studies was evaluated using the NOS score.

**Result:**

A total of 14 articles, including 3,890 patients, were included in the study, and the meta-analysis indicates that younger age in SLE patients [SMD = −0.23, 95% CI (−0.39, −0.06)], diabetes mellitus [OR = 1.78, 95% CI (1.03, 3.09)], hypertension [OR = 1.33, 95% CI (1.03, 1.72)], arthritis [OR = 1.57, 95% CI (1.24, 2.00)], Raynaud’s phenomenon [OR = 1.76, 95% CI (1.35, 2.30)], and cyclophosphamide use [OR = 2.24, 95% CI (1.38, 3.63)] are risk factors for the development of osteonecrosis in patients with SLE.

**Conclusion:**

The current study found that younger age, hypertension, diabetes, arthritis, Raynaud’s phenomenon, and cyclophosphamide use are independent risk factors for the development of osteonecrosis in patients with SLE.

## Introduction

Systemic lupus erythematosus (SLE) is a chronic, systemic autoimmune disease that affects multiple organs and systems (1, 2). It is common in young women and usually develops between the ages of 15 and 45 years (3). The clinical manifestations of SLE are varied and include, but are not limited to, rash, arthritis, renal damage, neurological damage, and cardiovascular lesions (4). The inflammatory response and tissue damage result from an abnormal immune system response to autologous cells. Epidemiological studies have shown that the prevalence of SLE varies across regions and ethnicities, but it is generally on the rise (5). With advances in therapeutic approaches, the survival of patients has been significantly improved, and the use of immunosuppressive therapy and anti-inflammatory drugs has significantly enhanced clinical outcomes (6). However, these treatments may also lead to various complications, such as osteoporosis and osteonecrosis, which can seriously affect patients’ quality of life and even endanger their lives (7, 8).

Osteonecrosis is a type of ischemic necrosis of bone tissue caused by an interruption of the blood supply, usually affecting large joints such as the femoral head and hip (9). The incidence of osteonecrosis is higher in patients with SLE, especially in patients on long-term steroid medication. Steroid therapy is a common treatment option for patients with SLE; however, its long-term use is strongly associated with the development of osteonecrosis (10, 11). The combination of osteonecrosis in patients with SLE significantly increases the risk of joint dysfunction, leading to severe pain, mobility problems, and, in some cases, the need for joint replacement surgery (12). Epidemiological studies have shown that the incidence of osteonecrosis is significantly higher in SLE patients than in the general population and that its early diagnosis and treatment are important (13). Nevertheless, the exact pathogenesis of osteonecrosis is still not fully understood and may involve a combination of factors (14). In SLE patients, osteonecrosis most frequently involves large joints such as the hip and knee; however, rare and atypical presentations may also occur. For example, Gökten (15) recently reported two cases of Freiberg’s infraction in patients with SLE, highlighting the potential for unusual skeletal sites to be affected and the importance of maintaining clinical vigilance beyond the classical locations.

This study aimed to assess the major risk factors for the development of osteonecrosis in patients with SLE through a systematic review and meta-analysis. Although there have been several individual studies (16, 17) examining the effects of factors such as steroid use, age, and gender on osteonecrosis in patients with SLE, no uniform conclusions have been reached due to conflicting findings and the small sample sizes of some studies. The present meta-analysis aims to pool high-quality existing studies to systematically assess various factors influencing the development of osteonecrosis in SLE patients, including treatment, genetics, immunity, and environment. Through this study, we aim to provide a more solid theoretical basis for the early prevention and treatment of osteonecrosis in SLE patients and to help clinicians in developing more effective, individualized treatment plans, thereby improving patients’ quality of life and long-term survival.

## Methods

### Literature search

The search was performed by searching PubMed, Embase, Web of Science, and Cochrane Library from database creation to 1 December 2024. The search terms were osteonecrosis, systemic lupus erythematosus, and risk factors, and the specific search strategy is described in Supplementary Table S1.

### Literature screening

Inclusion Criteria: This study included adults diagnosed with SLE, with osteonecrosis as the exposure factor and controls without osteonecrosis. The primary outcome was the identification of risk factors associated with the development of osteonecrosis. Only case–control or cohort studies were included.

Exclusion criteria: Studies were excluded if they were conference abstracts, meta-analyses, protocols, letters, previously published articles, systematic reviews, animal experiments, or if the full text or relevant data could not be obtained.

### Data extraction

Two independent evaluators screened the literature and extracted the data. Studies that could be readily accessed were directly screened by reviewing the title and abstract, followed by a full-text review when required. For studies that could be included with objections, we consulted relevant faculty members, directly downloaded and read the full texts, and strictly followed the inclusion and exclusion criteria during the screening process. We extracted the corresponding indicators from each study and performed cross-checks to ensure data consistency. The main data extracted included the first author’s name, publication year, study design, country, sample size, gender, age, and NOS scores. For studies where data were unavailable, we contacted the corresponding author, and if no data were received from them, such studies were excluded.

### Quality evaluation

The Newcastle–Ottawa Scale (NOS) (18) was used to evaluate the case–control study, including the selection of study population (4 points), comparability between groups (2 points), and measurement of exposure factors or results (3 points). The total score of the scale is 9, with ≤4 being low quality, 5–6 being medium quality, and ≥7 being high quality. If two researchers disagreed in the evaluation process, they discussed until a consensus was reached or a third party was asked to decide.

### Statistical analysis

Stata 15.0 was used for statistical data analysis. The risk values from each study were expressed as odds ratios (ORs), and the corresponding 95% confidence intervals were calculated. According to the results of the heterogeneity test (Q test) and the I^2^ statistic, the appropriate model was selected to calculate the combined RR value. If I^2^ was greater than 50%, a random-effects model was adopted; if I^2^ was less than or equal to 50%, a fixed-effects model was adopted. For cases where I^2^ > 50%, sensitivity analysis was performed using the leave-one-out method. Publication bias was assessed using Egger’s test, with a significance level *α* = 0.05. A *p*-value of < 0.05 was statistically significant.

## Results

### Literature search results

**Figure 1:**
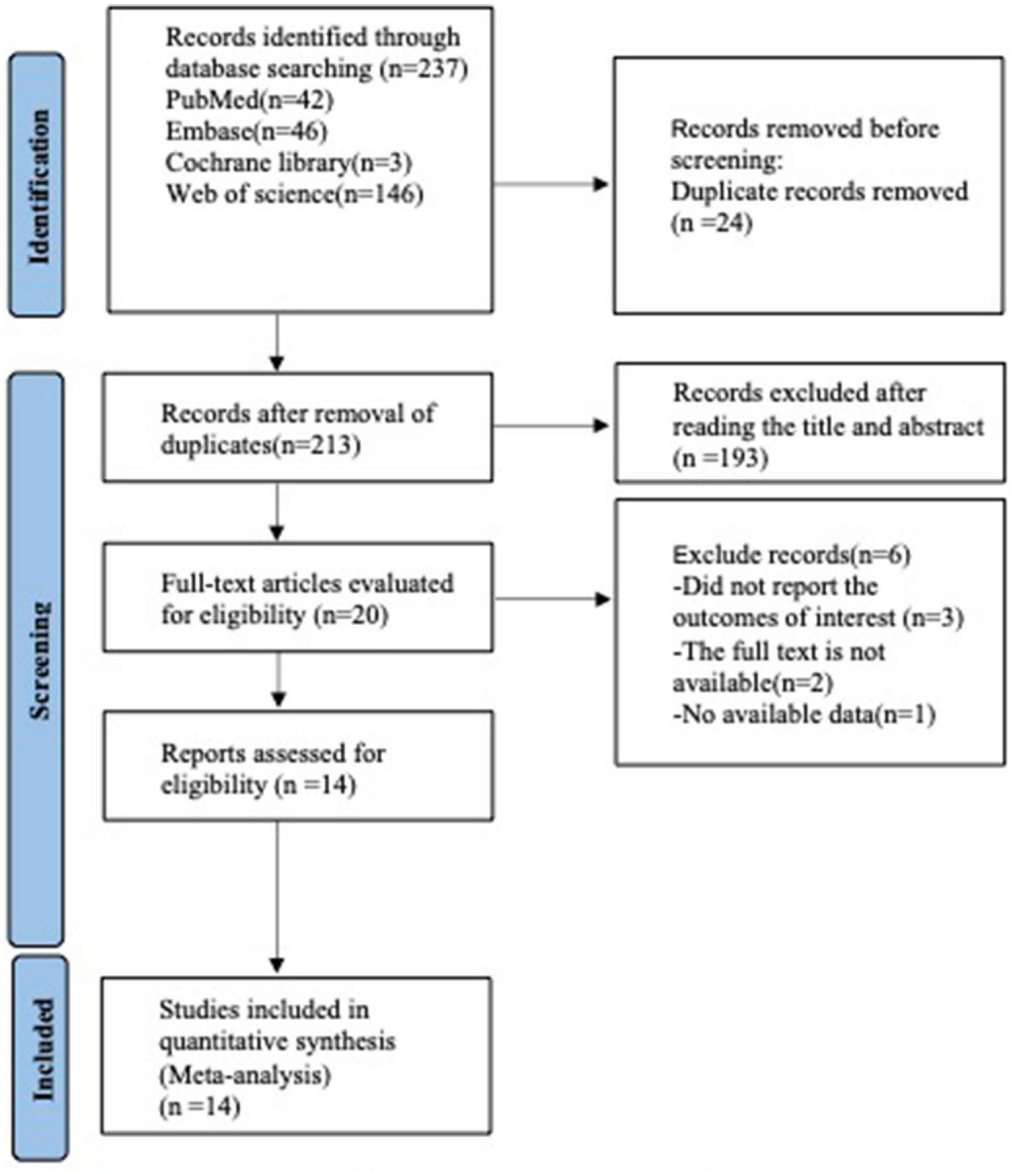
PRISMA flow diagram of the study process. PRISMA, preferred reporting items for systematic review and meta-analysis.

A preliminary search identified 42 studies from PubMed, 46 from Embase, 3 from Cochrane Library, and 146 from Web of Science. After removing duplicates (*n* = 23), excluding studies based on title and abstract screening (*n* = 193), and full-text review (*n* = 6), a total of 14 articles (19–31) were finally included in the analysis (see Figure 1).

### Basic characteristics of the included literature

A total of 14 articles, including 3,890 patients, were included, of which 2 were case–control studies and 12 were cohort studies. This study was scored using the NOS score, and all studies received scores of 7 or more. The specific basic characteristics are shown in Table 1.

**Table 1:** Basic characteristics.

Study	Year	Study design	Country	sample size	Gender (M/F)	Mean age (years)	NOS
Calvo	2006	Case–control	USA	91	17/74	35.5	8
Castro	2011	Cohort study	USA	40	7/33	11.2	9
Fialho	2007	Cohort study	Brazil	46	0/46	34.1	9
Hamza	2019	Cohort study	Egypt	60	5/55	32.2	8
Kuroda	2015	Cohort study	Japan	78	8/70	32.4	7
Lee	2014	Cohort study	Korea	128	12/116	23.4	8
Long	2021	Cohort study	China	1,158	63/1095	27.12	7
Nakamura	2010	Cohort study	Japan	131	NA	25.4	7
Sayarlioglu	2012	Cohort study	Turkey	203	24/179	28.7	8
Xu	2024	Cohort study	China	793	0/793	35.46	9
Zhao	2016	Case–control	China	100	NA	34.67	7
ZY Liu	2011	Cohort study	China	80	11/69	31.2	7
WB Xu	2024	Cohort study	China	914	87/827	30.03	7
X Wang	2024	Cohort study	China	68	8/60	32.1	7

## Results of meta-analysis

### Age

**Figure 2:**
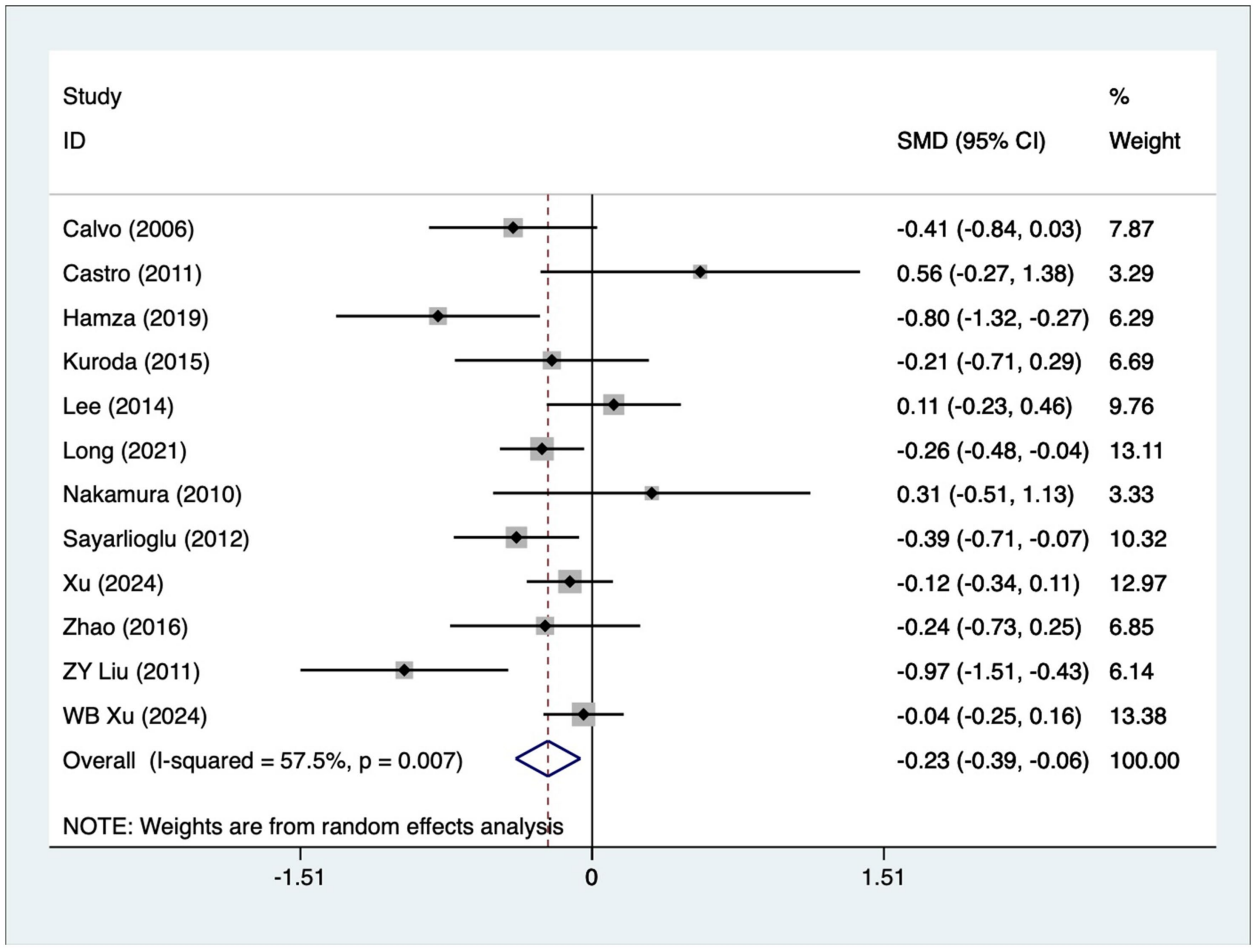
Forest plot of age meta-analysis.

A total of 12 articles reported patient age. A heterogeneity test (I^2^ = 57.5%, *p* = 0.007) was performed, and a random-effects model was used for the analysis. The results (Figure 2) indicate that younger SLE patients are more likely to develop osteonecrosis [SMD = −0.23, 95% CI (−0.39, −0.06)]. Due to high heterogeneity, a sensitivity analysis was performed by sequentially excluding each study. The results (Supplementary Figure S1) demonstrated low sensitivity and confirmed the stability of the analysis results.

### Diabetes

**Figure 3:**
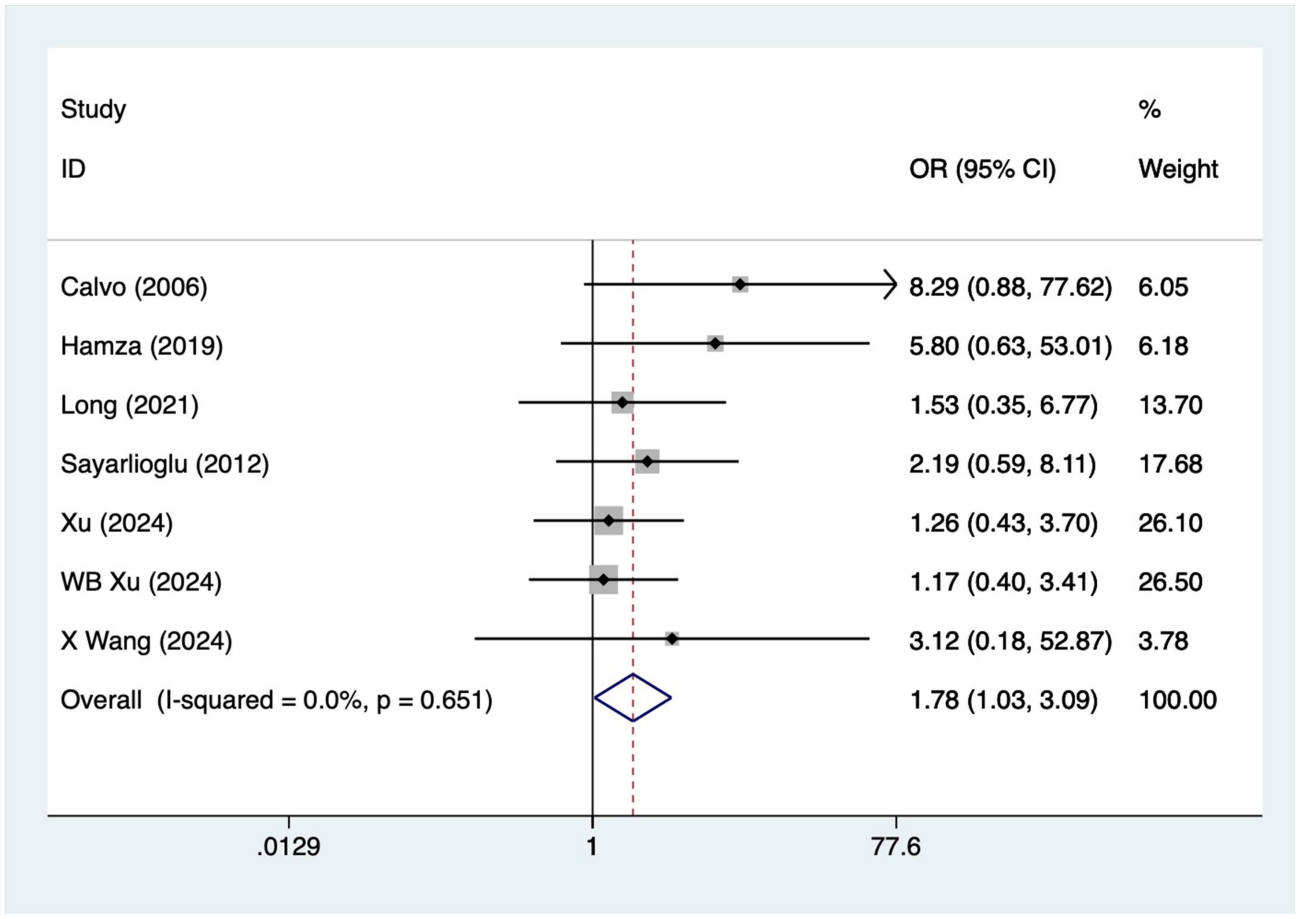
Forest plot of diabetes mellitus meta-analysis.

A total of seven articles reported on patients with diabetes mellitus. A heterogeneity test (I^2^ = 0%, *p* = 0.651) was performed using a fixed-effects model, and the analysis results (Figure 3) suggested that diabetes mellitus is a risk factor for the development of osteonecrosis in patients with SLE [OR = 1.78, 95% CI (1.03, 3.09)].

### Hypertension

**Figure 4:**
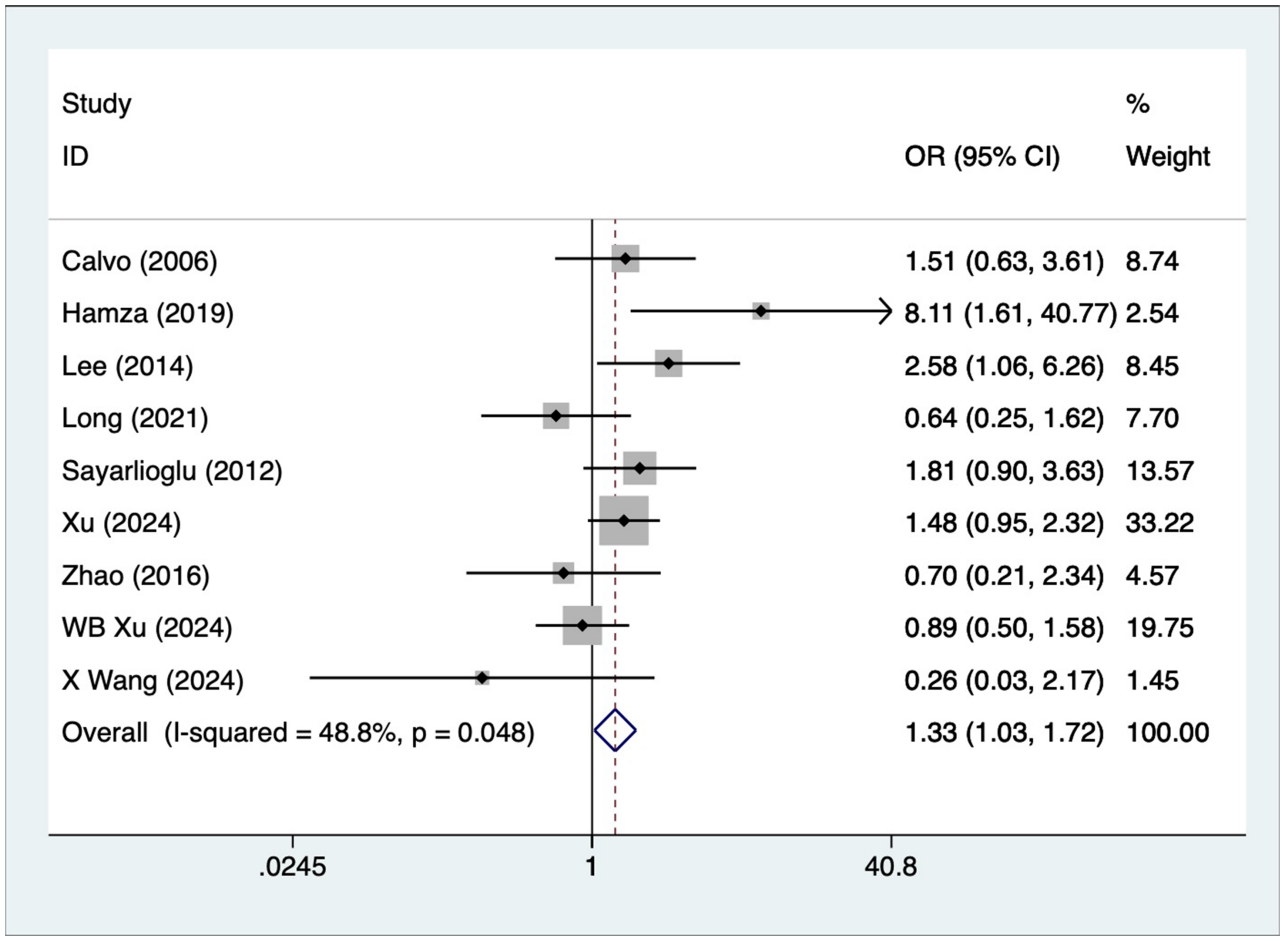
Forest plot of hypertension meta-analysis.

A total of nine articles reported on patients with hypertension. A heterogeneity test (I^2^ = 48.8%, *p* = 0.048) was performed using a fixed-effects model, and the analysis results (Figure 4) suggested that hypertension is a risk factor for the development of osteonecrosis in patients with SLE [OR = 1.33, 95% CI (1.03, 1.72)].

### Arthritis

**Figure 5:**
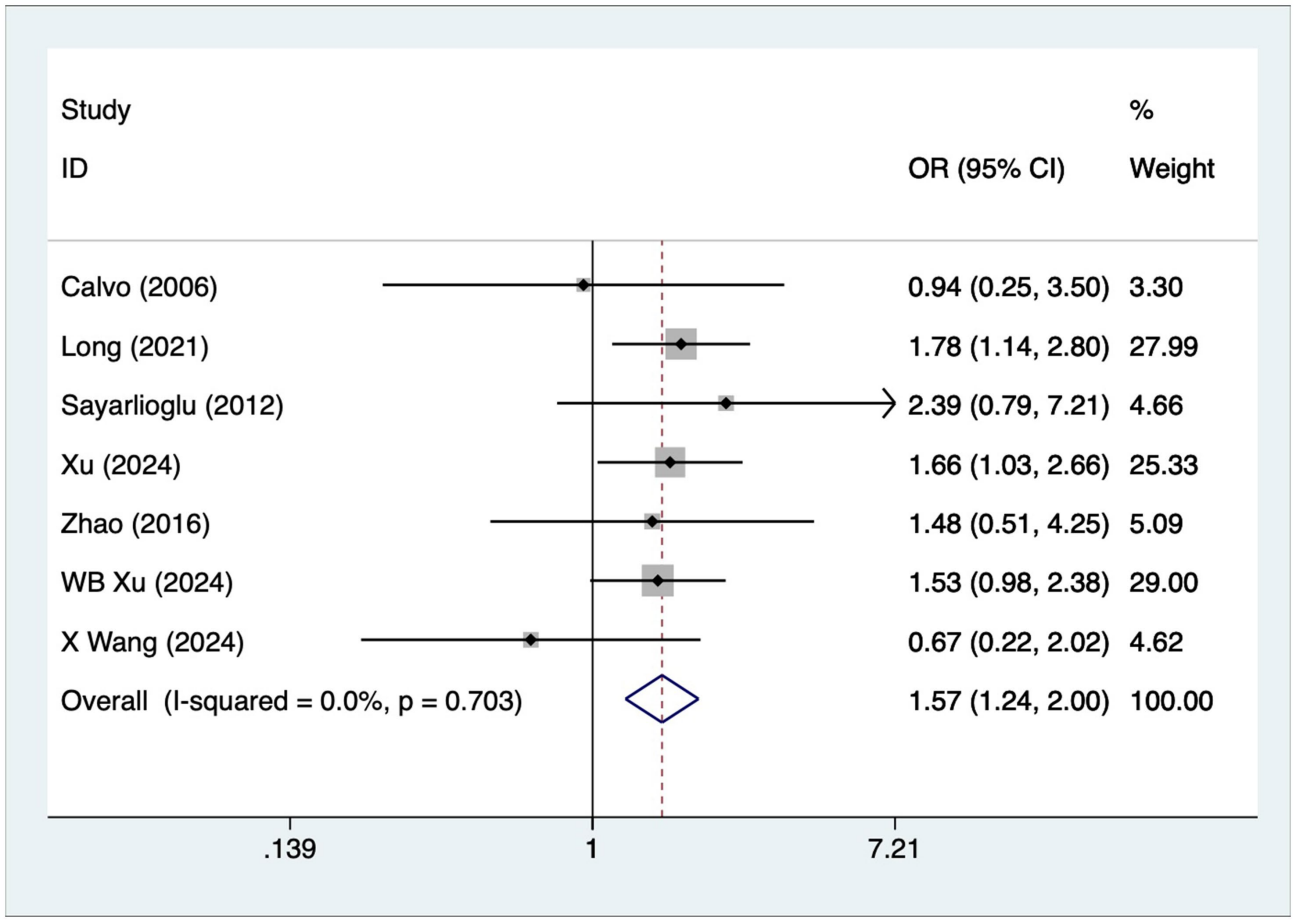
Forest plot of arthritis meta-analysis.

A total of seven articles reported on patients with arthritis. A heterogeneity test (I^2^ = 0%, *p* = 0.703) was performed using a fixed-effects model, and the analysis results (Figure 5) suggested that arthritis is a risk factor for the development of osteonecrosis in patients with SLE [OR = 1.57, 95% CI (1.24, 2.00)].

### Raynaud’s phenomenon

A total of eight articles reported on patients with Raynaud’s phenomenon. A heterogeneity test (I^2^ = 11%, *p* = 0.345) was performed using a fixed-effects model, and the analysis results (Supplementary Figure S2) suggested that Raynaud’s phenomenon is a risk factor for the development of osteonecrosis in patients with SLE [OR = 1.76, 95% CI (1.35, 2.30)].

### Cyclophosphamide

A total of five articles reported on patients with cyclophosphamide. A heterogeneity test (I^2^ = 68.5%, *p* = 0.013) was performed using a random-effects model, and the analysis results (Supplementary Figure S3) suggested that cyclophosphamide is a risk factor for the development of osteonecrosis in patients with SLE [OR = 2.24, 95% CI (1.38, 3.63)]. Due to high heterogeneity, sensitivity analysis was performed by sequentially excluding each study. The results (Supplementary Figure S4) suggest low sensitivity and stable analysis results.

### Publication bias

The present study used Egger’s test to assess publication bias, and the results (Supplementary Figures S5–S10) for age (0.677), diabetes mellitus (0.15), hypertension (0.91), arthritis (0.278), and Raynaud’s phenomenon (0.416) suggested no publication bias.

### Meta regression

Given the substantial heterogeneity observed in cyclophosphamide and age across studies, a meta-regression analysis was used to identify sources of heterogeneity. The meta-regression results (Table 2) indicate that study design, baseline treatments, and year of publication were not sources of heterogeneity between cyclophosphamide and age.

**Table 2:** Meta-regression results.

Cyclophosphamide				
Variable	Coef.	Std. Err	*p*	95%CI
Study design	0.138	0.133	0.318	[−0.147, 0.424]
Baseline treatments	0.016	0.117	0.894	[−0.236, 0.267]
Year of publication	0.012	0.014	0.429	[−0.196, 0.043]
age				
Study design	0.102	0.658	0.071	[−0.08, 0.2.89]
Baseline treatments	0.067	0.986	0.153	[−0.138 0.875]
Year of publication	0.186	0.753	0.099	[−0.354, 0.997]

## Discussion

To the best of our knowledge, this is the first meta-analysis to explore the risk factors for osteonecrosis in patients with SLE, and the results of the study identified young age, diabetes, hypertension, arthritis, Raynaud’s phenomenon, and cyclophosphamide as risk factors for osteonecrosis in patients with SLE.

Younger patients with SLE are more likely to develop osteonecrosis, a phenomenon that may be related to several mechanisms. First, young patients have active bone metabolism and relatively frequent bone remodeling processes, so when drugs such as glucocorticoids are used to treat SLE, the bone tissue in young patients is more susceptible to the effects of the drugs, leading to the disruption of the vascular supply to the bone, which in turn leads to osteonecrosis (32). In addition, young patients have a more active immune system, and immune complex deposition and vascular inflammation due to SLE may be more severe, further aggravating the risk of bone tissue damage and necrosis (33). Therefore, bone mineral density and bone metabolism indices should be monitored in young SLE patients, and effective interventions should be taken to reduce this complication and improve patients’ quality of life. The current study found that diabetes mellitus is a risk factor for osteonecrosis in patients with SLE. Mechanisms may include vascular lesions and microcirculatory disturbances caused by diabetes, toxic effects of hyperglycemia on bone cells, chronic inflammation exacerbated by abnormalities in the immune system, and the occlusion of bone vessels due to lipid metabolism disorders (34). This conclusion is supported by similar studies showing a significantly higher incidence of osteonecrosis in diabetic patients than in non-diabetic patients (35). Preventive measures, including tight control of blood glucose levels and regular monitoring of blood glucose and glycated hemoglobin, may be effective in reducing the risk of diabetes-related osteonecrosis in patients with SLE (36). The current study suggests that hypertension is a risk factor for osteonecrosis in patients with SLE. The mechanisms may include hypertension-induced vascular endothelial dysfunction and microcirculatory disorders, and these lesions lead to reduced blood supply to bone tissue, increasing the risk of osteonecrosis. In addition, patients with hypertension are often associated with atherosclerosis, which may also exacerbate the lack of blood flow and hypoxia to bone tissue (37, 38). For hypertensive SLE patients, special attention should be paid to avoiding high-dose and long-term use of glucocorticoids and taking calcium supplementation, which can effectively reduce the risk of hypertension-related osteonecrosis among SLE patients. Arthritis is a risk factor for osteonecrosis in patients with SLE. The mechanisms may include inflammatory responses and immune system abnormalities due to arthritis, and these changes affect the blood supply and metabolic function of bone tissue, increasing the risk of osteonecrosis (39). Timely calcium and vitamin D supplementation, appropriate physical activity, and avoidance of smoking and excessive alcohol consumption are also important preventive tools (40). Through these comprehensive measures, the risk of arthritis-related osteonecrosis can be effectively reduced among SLE patients. Raynaud’s phenomenon is a risk factor for osteonecrosis in patients with SLE. It causes vasospasm and microcirculatory disturbances that reduce the blood supply to bone tissue and increase the risk of osteonecrosis (41). Preventive measures, including the control of Raynaud’s phenomenon, regular monitoring of vascular health, and reduction of glucocorticoid use, can effectively reduce the risk of Raynaud’s phenomenon-related osteonecrosis in SLE patients. Cyclophosphamide is a risk factor for osteonecrosis in patients with SLE. Cyclophosphamide is a commonly used immunosuppressant, primarily for the treatment of SLE and other autoimmune diseases (42). However, its use may increase the risk of osteonecrosis by mechanisms that may include drug-induced vascular endothelial damage and bone marrow suppression, leading to reduced blood supply and decreased repair capacity of bone tissue (43). Minimizing the dose and duration of cyclophosphamide use, choosing other immunosuppressant alternatives, strengthening the monitoring of patients’ bone health, and performing regular bone density examinations are measures that may help to reduce the risk of cyclophosphamide-associated osteonecrosis in patients with SLE.

As a recognized risk factor for osteonecrosis in SLE, glucocorticoids have been confirmed in numerous studies. However, we could not quantitatively analyze it due to insufficient data on the associated steroid use in this study. Future studies should further collect and analyze relevant data to assess the specific effects of glucocorticoids on osteonecrosis in SLE. For age, heterogeneity may be partly explained by the lack of a standardized definition of “younger” patients, with some studies using a threshold of <30 years, while others used <40 years or alternative cut-offs. For cyclophosphamide, differences in treatment protocols—including cumulative dose, duration of exposure, and route of administration—likely contributed to variability in the results.

### Clinical implications

Based on the results of this meta-analysis, clinicians can effectively prevent osteonecrosis in patients with SLE by identifying high-risk individuals and implementing early interventions. First, younger patients, particularly those with SLE at a younger age, are more susceptible to developing osteonecrosis. Therefore, regular imaging screenings—especially MRI or CT scans—are essential for early detection and treatment. Additionally, diabetes, hypertension, and arthritis represent significant risk factors. Clinicians should actively monitor these comorbidities, maintain optimal glycemic and blood pressure control, and promptly treat arthritis symptoms to reduce osteonecrosis incidence. For patients receiving cyclophosphamide, the necessity of its use should be carefully evaluated, and alternative immunosuppressive agents should be considered to mitigate osteonecrosis risk. Regular examinations, particularly in high-risk patients, enable the early detection of osteonecrosis. This allows for timely intervention, such as combined physical therapy and medication, thereby improving patient prognosis. Overall, comprehensive management of risk factors in SLE patients, combined with individualized treatment plans, is key to preventing osteonecrosis.

This meta-analysis has several limitations that should be acknowledged. First, although 14 studies were included overall, the number of studies contributing to some individual factors was relatively small (e.g., five for cyclophosphamide and seven for diabetes). This limited evidence base reduces the statistical power and increases the risk of type II error, and therefore, the corresponding findings should be interpreted with caution. Second, moderate-to-high heterogeneity was observed in certain analyses, particularly for age (I^2^ = 57.5%) and cyclophosphamide (I^2^ = 68.5%). Such heterogeneity may stem from differences in patient populations (e.g., ethnicity, baseline disease activity, and comorbidities) as well as variations in treatment regimens, including cumulative drug dose, administration route, and concomitant therapies. Although sensitivity analyses suggested that the results were relatively stable, the presence of heterogeneity indicates that these findings may not be uniformly applicable across all clinical settings. Third, most included studies only reported univariate data, and our analyses were therefore based on unadjusted odds ratios. This prevents adequate control for confounding factors such as disease severity, treatment history, and comorbid conditions. For instance, the observed association between cyclophosphamide use and osteonecrosis may be partly explained by confounding by indication, since the drug is more frequently administered to patients with severe systemic lupus erythematosus. Furthermore, unmeasured confounders are likely to have influenced our results, as most of the included studies were observational in nature. These confounding factors could include disease activity, cumulative steroid dose, and the use of concomitant immunosuppressants, all of which may have an independent effect on the development of osteonecrosis. The absence of these variables in the analyses limits the ability to draw definitive conclusions about the independent impact of the identified risk factors. Taken together, these limitations suggest that the results should be viewed as exploratory and hypothesis-generating rather than definitive. Further large-scale, high-quality prospective studies using multivariable analyses that can account for these confounders are needed to confirm whether the identified factors represent independent risk determinants.

## Conclusion

The current study found that young age, hypertension, diabetes, arthritis, Raynaud’s phenomenon, and cyclophosphamide use were identified as independent risk factors for the development of osteonecrosis in patients with SLE. However, because of the heterogeneity of the studies, we need more high-quality, multicenter, large-sample studies to justify our conclusions.

## Data Availability

The original contributions presented in the study are included in the article/Supplementary material, further inquiries can be directed to the corresponding author.
