# Supplementary material for: Risk factors of osteonecrosis in patients with systemic lupus erythematosus: a meta-analysis
Source: Front Med (Lausanne). 2025 Oct 17;12:1694721. doi: 10.3389/fmed.2025.1694721 (PMC12575097; doi:10.3389/fmed.2025.1694721)
Supplement: Supplementary file 1 [file Data_Sheet_1.docx]

Table s1 Search strategy

((("Lupus Erythematosus, Systemic"[Mesh]) OR ((((((Lupus Erythematosus, Systemic[Title/Abstract]) OR (Lupus Erythematosus Disseminatus[Title/Abstract])) OR (Systemic Lupus Erythematosus[Title/Abstract])) OR (Libman-Sacks Disease[Title/Abstract])) OR (Disease, Libman-Sacks[Title/Abstract])) OR (Libman Sacks Disease[Title/Abstract]))) AND (("Femur Head Necrosis"[Mesh]) OR (((((((((((Femur Head Necrosis[Title/Abstract]) OR (Femur Head Necroses[Title/Abstract])) OR (Head Necrosis, Femur[Title/Abstract])) OR (Necrosis, Femur Head[Title/Abstract])) OR (Necrosis, Avascular, of Femur Head[Title/Abstract])) OR (Avascular Necrosis Of Femoral Head, Primary[Title/Abstract])) OR (Avascular Necrosis of Femur Head[Title/Abstract])) OR (Femoral Head, Avascular Necrosis Of[Title/Abstract])) OR (Ischemic Necrosis Of Femoral Head[Title/Abstract])) OR (Aseptic Necrosis of Femur Head[Title/Abstract])) OR (Necrosis, Aseptic, of Femur Head[Title/Abstract])))) AND (("Risk Factors"[Mesh]) OR (((((((((((((((((((Risk Factors[Title/Abstract]) OR (Factor, Risk[Title/Abstract])) OR (Risk Factor[Title/Abstract])) OR (Population at Risk[Title/Abstract])) OR (Populations at Risk[Title/Abstract])) OR (Risk Scores[Title/Abstract])) OR (Risk Score[Title/Abstract])) OR (Score, Risk[Title/Abstract])) OR (Risk Factor Scores[Title/Abstract])) OR (Risk Factor Score[Title/Abstract])) OR (Score, Risk Factor[Title/Abstract])) OR (Health Correlates[Title/Abstract])) OR (Correlates, Health[Title/Abstract])) OR (Social Risk Factors[Title/Abstract])) OR (Factor, Social Risk[Title/Abstract])) OR (Factors, Social Risk[Title/Abstract])) OR (Risk Factor, Social[Title/Abstract])) OR (Risk Factors, Social[Title/Abstract])) OR (Social Risk Factor[Title/Abstract])))


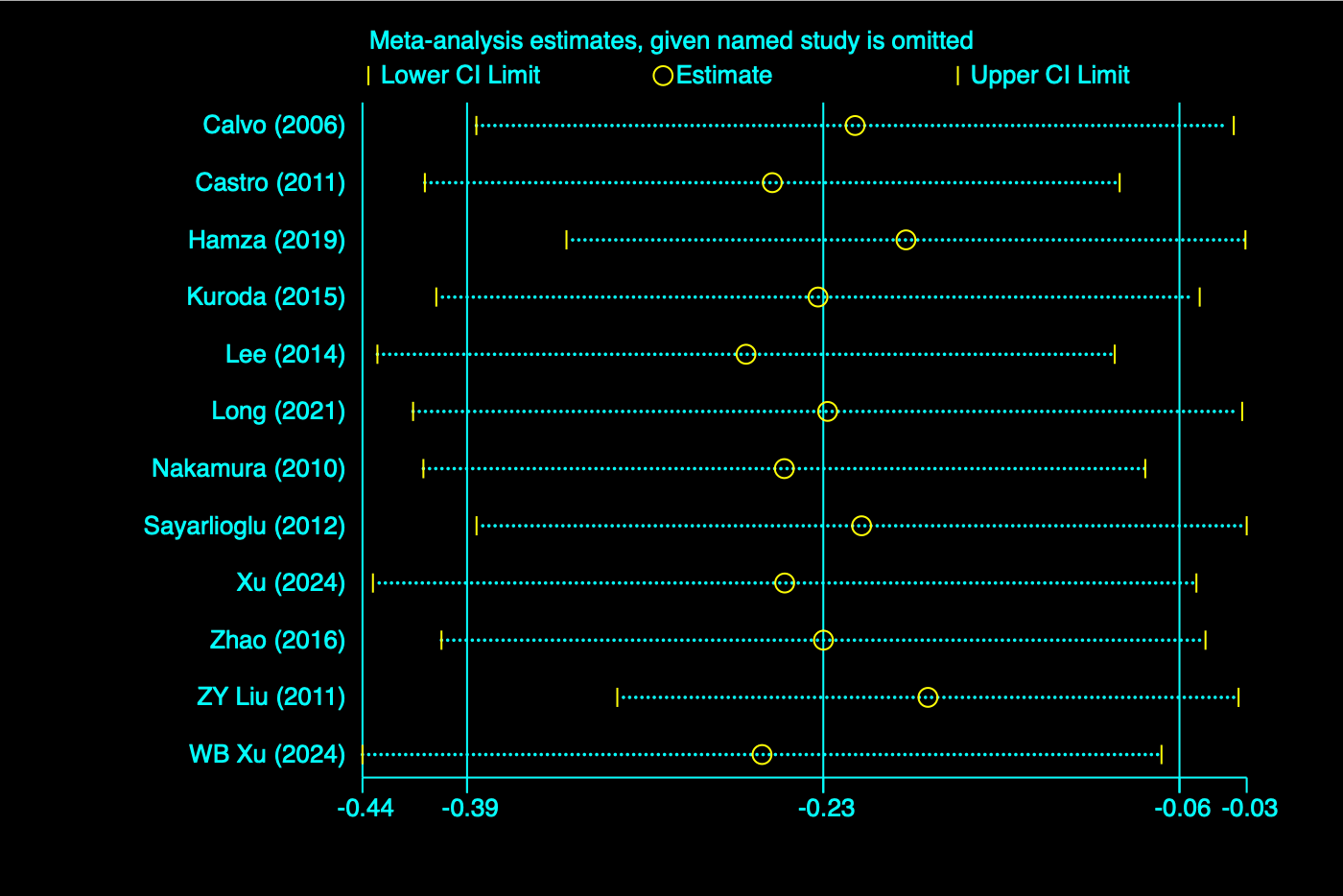


Figure s1 age sensitivity analysis


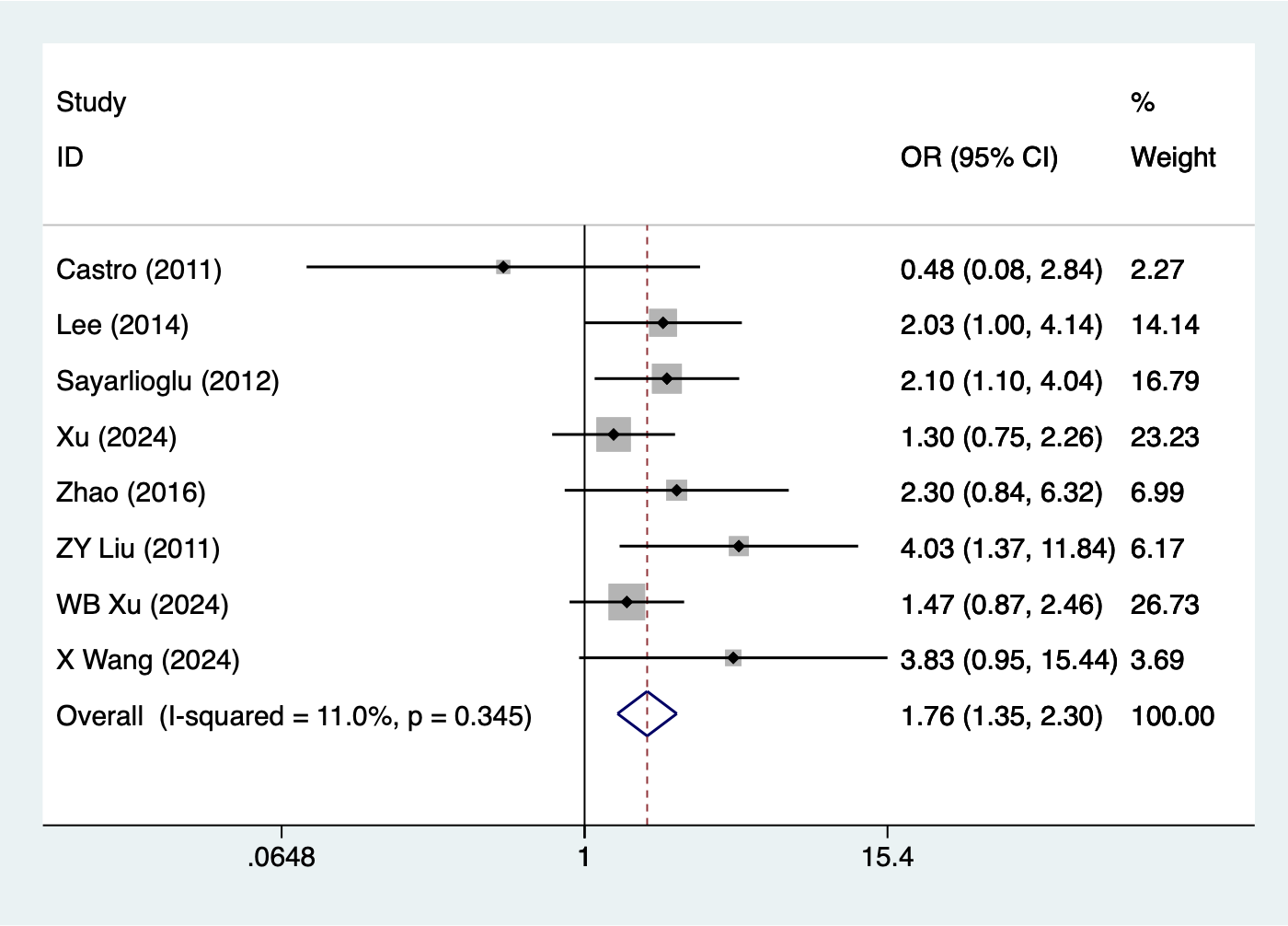


Figure S2 Forest plot of Raynaud's phenomenon meta-analysis


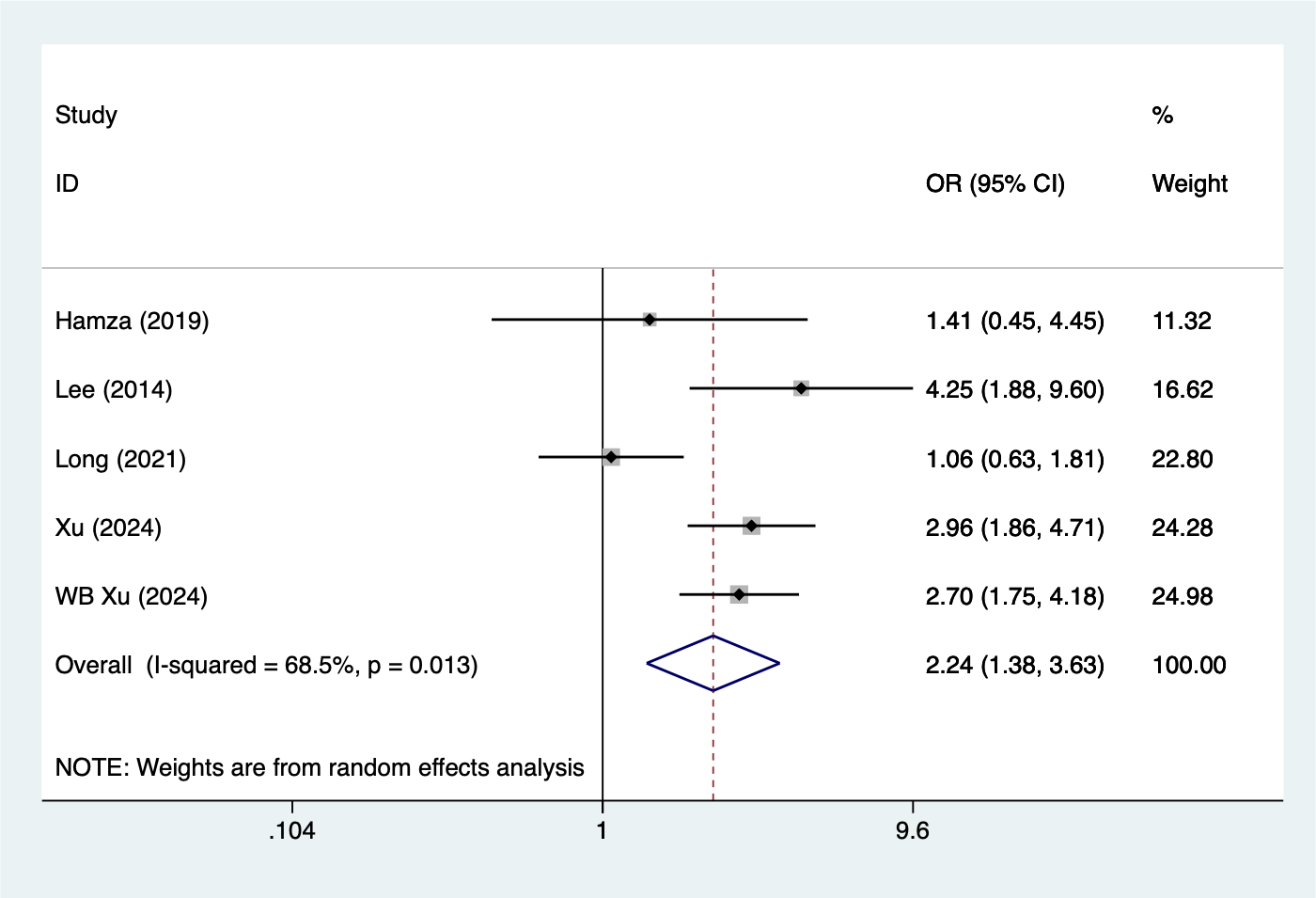


Figure s3 Forest plot of cyclophosphamide meta-analysis


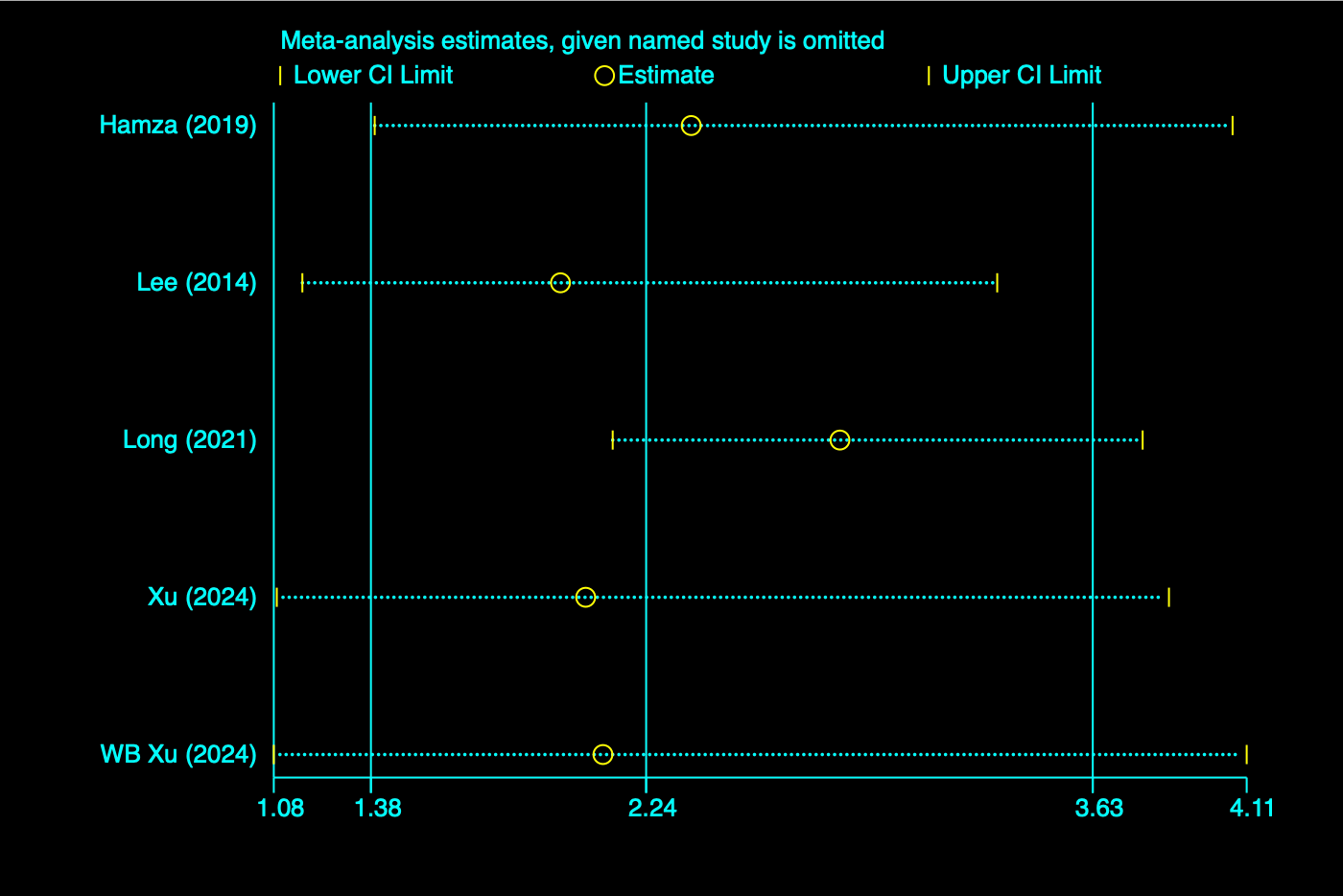


Figure s4 cyclophosphamide sensitivity analysis


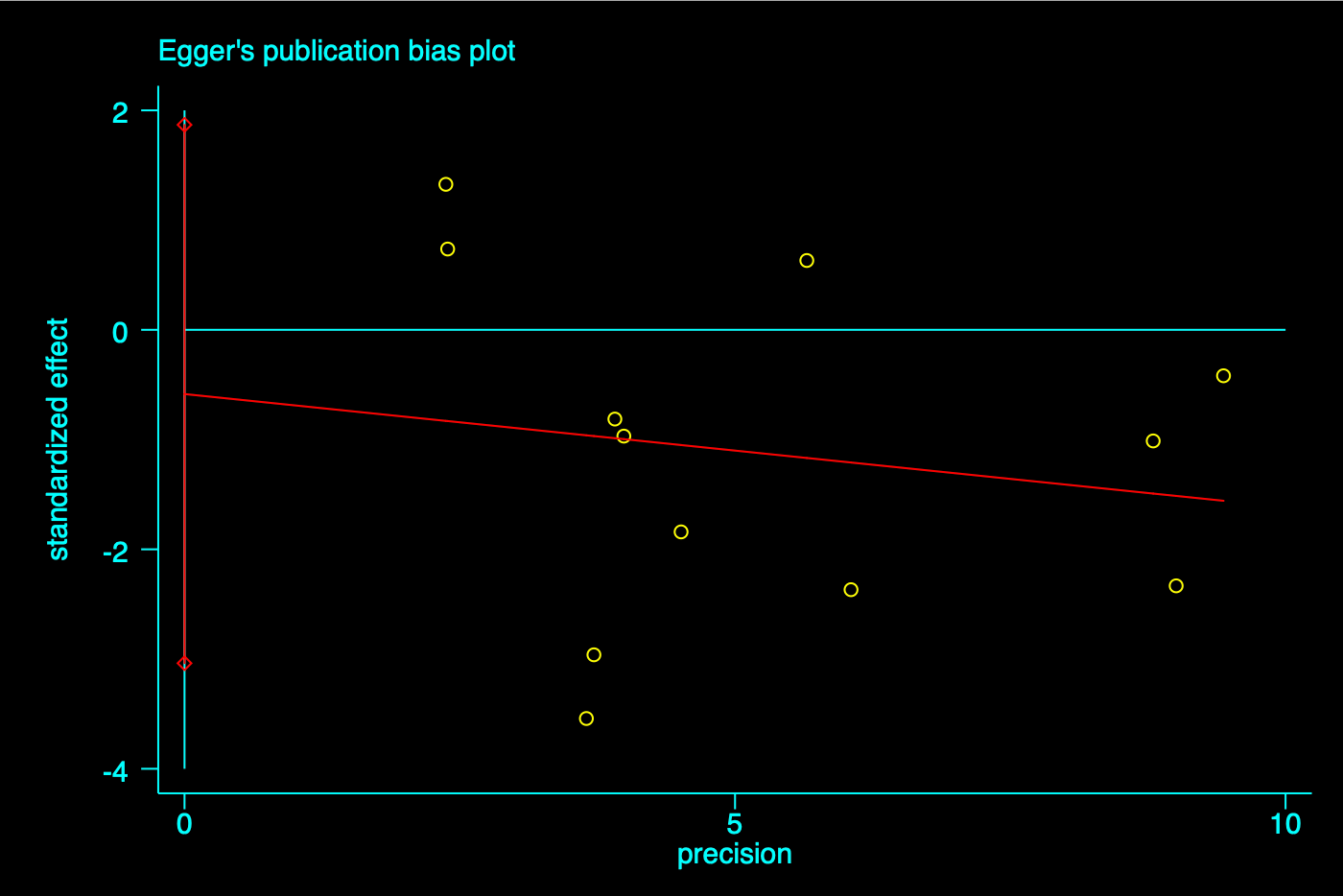


Figure s5 age egger test


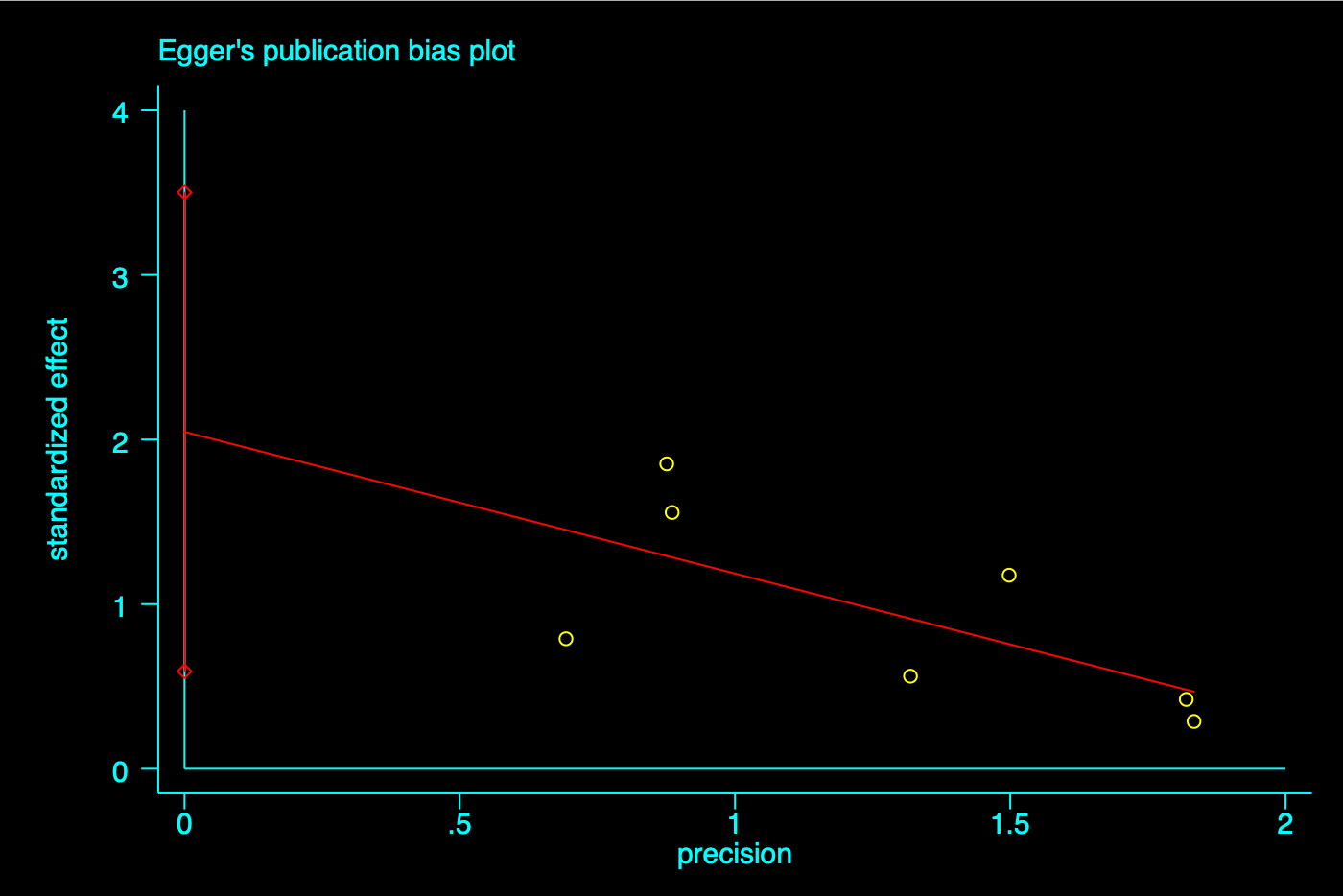


Figure s6 diabetes mellitus egger test


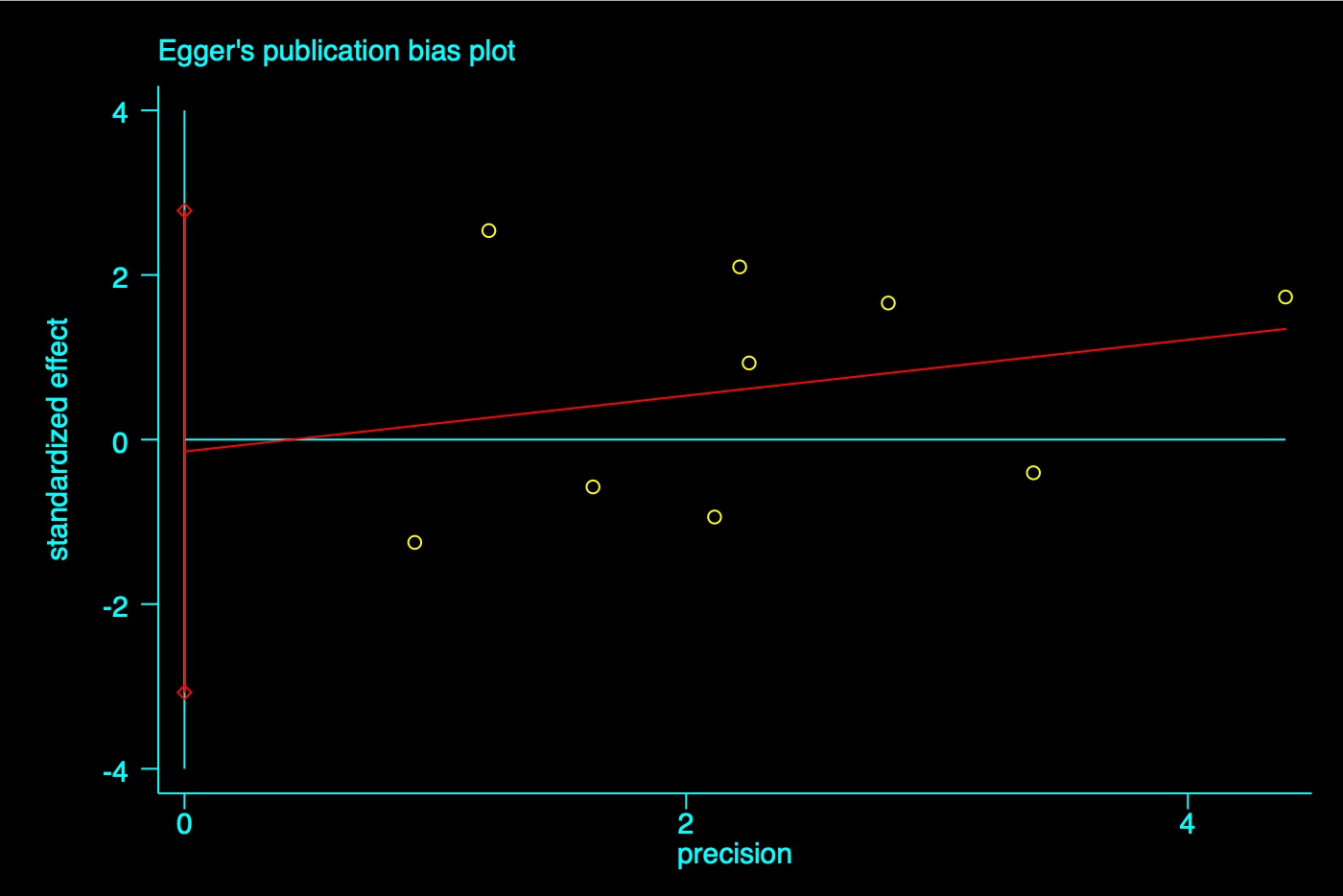


Figure s7 hypertension egger test


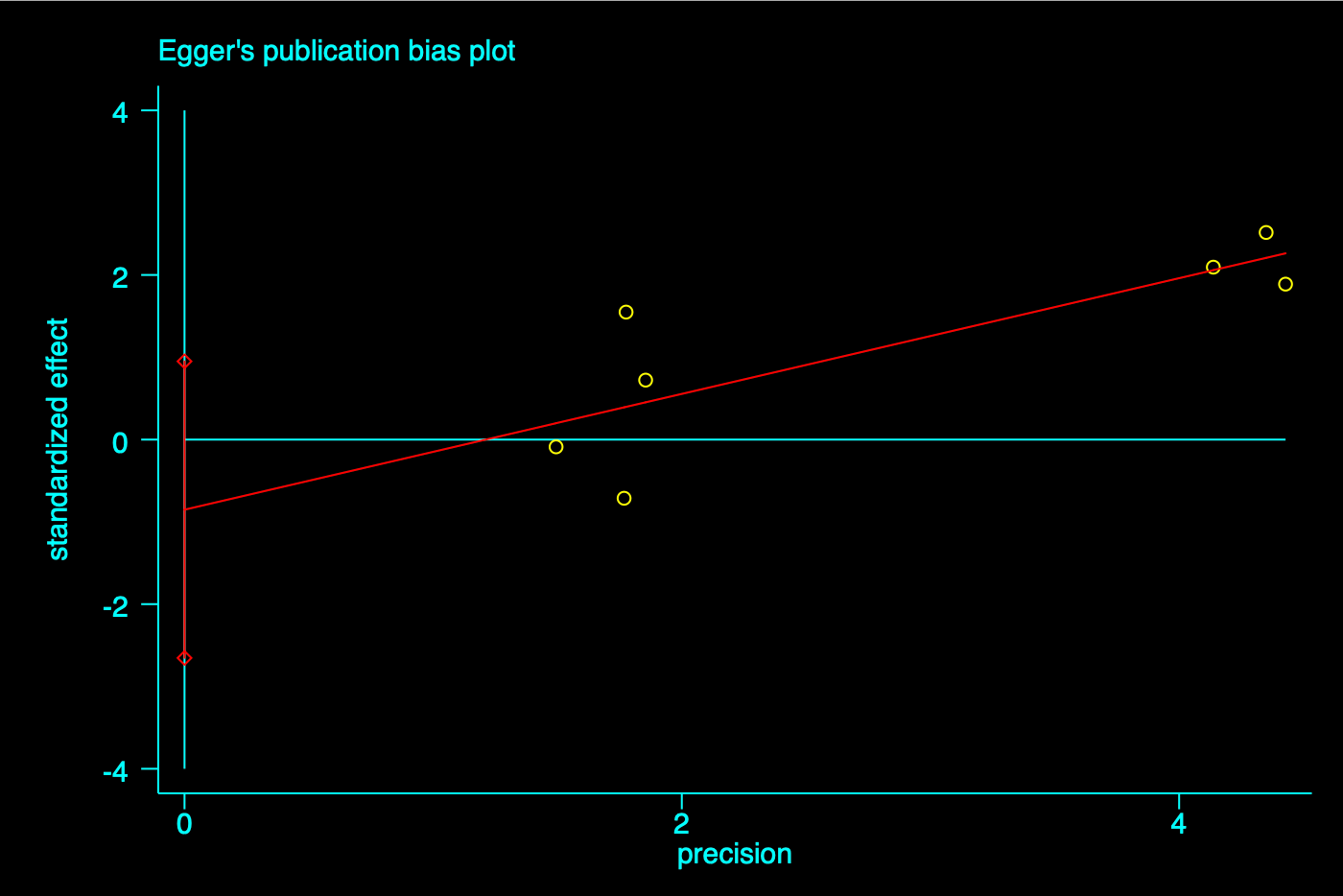


Figure s8 Arthritis egger test


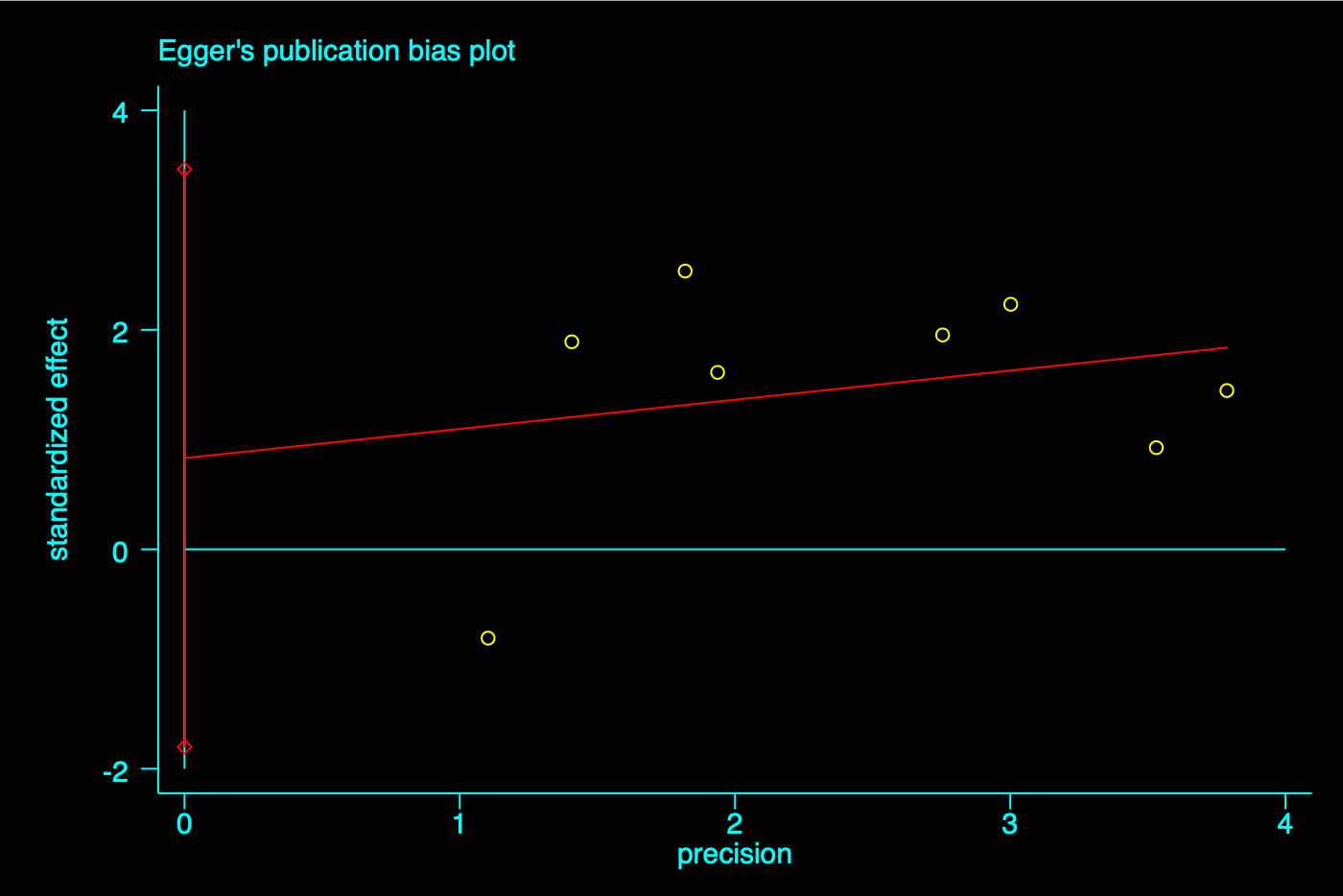


Figure s9 Raynaud’s phenomenon egger test


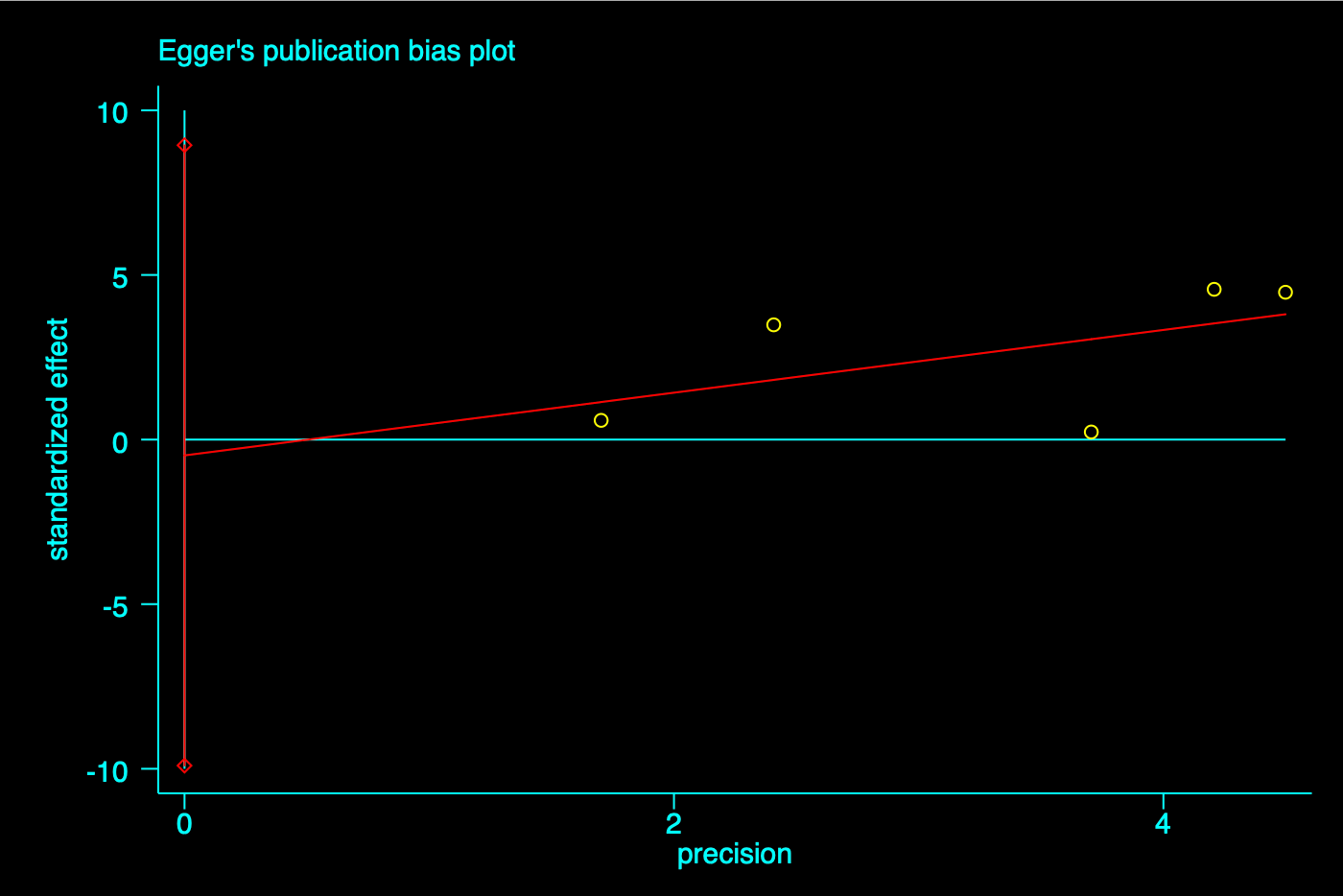


Figure s10 cyclophosphamide egger test
